# Dietary Acrylamide Intake during Pregnancy and Fetal Growth—Results from the Norwegian Mother and Child Cohort Study (MoBa)

**DOI:** 10.1289/ehp.1205396

**Published:** 2012-11-29

**Authors:** Talita Duarte-Salles, Hans von Stedingk, Berit Granum, Kristine B. Gützkow, Per Rydberg, Margareta Törnqvist, Michelle A. Mendez, Gunnar Brunborg, Anne Lise Brantsæter, Helle Margrete Meltzer, Jan Alexander, Margaretha Haugen

**Affiliations:** 1 Division of Environmental Medicine, Norwegian Institute of Public Health, Oslo, Norway; 2 Department of Materials and Environmental Chemistry, Arrhenius Laboratory, Stockholm University, Sweden; 3 Department of Nutrition, Gillings School of Global Public Health, University of North Carolina at Chapel Hill, Chapel Hill, North Carolina, USA; 4 Office of Director-General, Norwegian Institute of Public Health, Oslo, Norway

**Keywords:** acrylamide, birth weight, diet, Hb adducts, MoBa, pregnancy, small for gestational age

## Abstract

Background: Acrylamide has shown developmental and reproductive toxicity in animals, as well as neurotoxic effects in humans with occupational exposures. Because it is widespread in food and can pass through the human placenta, concerns have been raised about potential developmental effects of dietary exposures in humans.

Objectives: We assessed associations of prenatal exposure to dietary acrylamide with small for gestational age (SGA) and birth weight.

Methods: This study included 50,651 women in the Norwegian Mother and Child Cohort Study (MoBa). Acrylamide exposure assessment was based on intake estimates obtained from a food frequency questionnaire (FFQ), which were compared with hemoglobin (Hb) adduct measurements reflecting acrylamide exposure in a subset of samples (*n* = 79). Data on infant birth weight and gestational age were obtained from the Medical Birth Registry of Norway. Multivariable regression was used to estimate associations between prenatal acrylamide and birth outcomes.

Results: Acrylamide intake during pregnancy was negatively associated with fetal growth. When women in the highest quartile of acrylamide intake were compared with women in the lowest quartile, the multivariable-adjusted odds ratio (OR) for SGA was 1.11 (95% CI: 1.02, 1.21) and the coefficient for birth weight was –25.7 g (95% CI: –35.9, –15.4). Results were similar after excluding mothers who smoked during pregnancy. Maternal acrylamide– and glycidamide–Hb adduct levels were correlated with estimated dietary acrylamide intakes (Spearman correlations = 0.24; 95% CI: 0.02, 0.44; and 0.48; 95% CI: 0.29, 0.63, respectively).

Conclusions: Lowering dietary acrylamide intake during pregnancy may improve fetal growth.

Indicators of intrauterine development, such as birth weight and small for gestational age (SGA), are predictors of infant survival and the prevalence of chronic diseases in adulthood (Barker 2002; Risnes et al. 2011). Diet during pregnancy is a well-recognized determinant of fetal growth (Godfrey and Barker 2000). A decade ago it was shown that acrylamide is formed during heating of food at high temperatures, and that it is found in a variety of foods (Dybing et al. 2005; Tareke et al. 2000, 2002). Acrylamide has been in industrial use since the 1950s and is also present in cigarette smoke (Bergmark 1997; Shipp et al. 2006). Acrylamide has shown neurotoxic effects in humans with occupational exposures (Hagmar et al. 2001) and is classified as a probable human carcinogen (International Agency for Research on Cancer 1994). Its metabolite glycidamide is assumed to be the genotoxic agent of acrylamide (Rice 2005). Acrylamide is known to induce developmental and reproductive toxicity in animals including effects on fetal growth (Manson et al. 2005).

In humans, fetal exposure to acrylamide through the diet may start *in utero*, because acrylamide has been found to cross the placenta barrier *in vitro* (Annola et al. 2008; Sörgel et al. 2002) as well as *in vivo* (Schettgen et al. 2004; von Stedingk et al. 2011). Recently, we showed that higher acrylamide exposure among nonsmoking pregnant women was associated with reduced fetal growth based on birth weight and SGA (Pedersen et al. 2012). That study included 1,101 women from five different European countries, and the exposure assessment was based on acrylamide hemoglobin (Hb) adduct levels measured in cord blood, as well as food scores created from acrylamide-rich food intakes obtained from food frequency questionnaires (FFQ).

Dietary acrylamide exposure estimated from FFQ data used in the The Norwegian Mother and Child Cohort Study (MoBa) has previously been validated using urine metabolites as biomarker of recent intakes (Brantsaeter et al. 2008b). N-terminal Hb adducts reflect longer time window for exposure (approximately 120 days circulation time for erythrocytes) compared with urine metabolites (just a few days), and have been used for biomonitoring acrylamide exposure in many studies (e.g., Törnqvist et al. 2006). The validity of Hb adducts as a marker of acrylamide exposure from food has been demonstrated in animal studies as well as in human intervention studies (Abramsson-Zetterberg et al. 2008; Naruszewicz et al. 2009). In other studies comparing FFQ-based acrylamide intake estimates with measured acrylamide–Hb adduct concentrations low to moderate correlations were observed (Bjellaas et al. 2007; Kütting et al. 2008; Tran et al. 2010; Wilson et al. 2009a, 2009b; Wirfält et al. 2008).

In this study we explored the hypothesis that dietary acrylamide exposure during prenatal life might impair fetal growth, resulting in increased SGA and reduced birth weight in a large population-based cohort study in Norway: MoBa. We also aimed to identify population characteristics associated with higher intakes of acrylamide. The FFQ-based estimates of acrylamide intake were further evaluated by comparisons with measurements of acrylamide– and glycidamide–Hb adducts in a subset of the study participants.

## Methods

*Population and study design*. MoBa is a prospective population-based pregnancy cohort study conducted by the Norwegian Institute of Public Health (Magnus et al. 2006). Participants were recruited from all over Norway during 1999–2008, and 38.5% of invited women consented to participate. The cohort now includes 108,000 children, 90,700 mothers, and 71,500 fathers. Blood samples were obtained from both parents during pregnancy and from mothers and children (umbilical cord) at birth. Follow-up is conducted by questionnaires at regular intervals and by linkage to national health registries. Several substudies are conducting additional collections of data and biological materials. The present study is based on version 5 of the quality-assurance data files released for research in June 2010 (unpublished data). Informed consent was provided by each MoBa participant upon recruitment. The study was approved by the Regional Committee of Medical Research Ethics for South-Eastern Norway.

Women were eligible for the present analysis if they were recorded in the Medical Birth Registry of Norway (MBRN) and had singleton births, and they completed questionnaires 1 and 3 (in weeks 17 and 30 of pregnancy, respectively), baseline MoBa questionnaires covering information on sociodemographic characteristics, exposure to tobacco smoke during pregnancy, and general health; questionnaire 2 (during weeks 23–24 of pregnancy), which covered dietary information; and questionnaire 4 (when the child was 6 months of age), which collected information on maternal health at time of delivery, including maternal weight gain during pregnancy (*n* = 62,124) (Norwegian Institute of Public Health 2007). We then excluded women if they participated in MoBa with multiple pregnancies (*n* = 6,604), if gestational age at the child’s birth was < 28 weeks or > 42 weeks (*n* = 385), if data were missing on birth weight (*n* = 22) or maternal smoking during pregnancy (*n* = 817), or if the mother’s estimated energy intake was < 4,500 kJ or > 20,000 kJ (*n* = 796). In addition, we excluded women with missing (*n* = 2,386) or improbable values for weight gain during pregnancy (< –30 kg or > 50 kg) (*n* = 463), leaving a study sample of 50,651 women. Because smoking is negatively associated with birth weight and is a significant source of acrylamide exposure, we performed additional analyses stratified on self-reported smoking during pregnancy (46,420 nonsmokers and 4,231 smokers).

*Dietary information*. The MoBa FFQ (MoBa 2002) was used for calculation of acrylamide intake. This FFQ is a semiquantitative questionnaire designed to provide information on dietary habits and dietary supplement intakes during the first 4–5 months of pregnancy (Meltzer et al. 2008). It has been thoroughly validated with regard to foods and nutrients (Brantsaeter et al. 2008a). For each of the 255 food and beverage items, the frequency of consumption was reported by selecting one of 8–10 possible frequencies, ranging from never to several times monthly, weekly, or daily. Energy intake was calculated from the FFQ using FoodCalc (Lauritsen 2005) and the Norwegian Food Composition table (Rimestad et al. 2005).

To calculate acrylamide intake, we prepared a database containing values of acrylamide concentration reported from analyses of Norwegian food items (Norwegian Food Safety Authority 2002, 2006; Scientific Committee of the Norwegian Food Safety Authority 2002) and the Swedish National Food Administration (Livsmedelsverket 2002). For foods not analyzed in Norway or Sweden, we collected data from the European Union database (Institute for Reference Materials and Measurements 2005). For food items with multiple analyses of acrylamide concentration, the median concentration was used. Examples of the values assigned for each food group have previously been published (Brantsaeter et al. 2008b). To identify food group predictors of higher acrylamide intake, the 255 food items in the FFQ were grouped into 19 food groups based on nutrient profiles, culinary usage, or known acrylamide levels. For example, cereals and potatoes were classified into four separate food groups: fried potatoes; crisp bread; bread, which included dark and white bread; and other, including breakfast cereal, rice, couscous, pasta, and pizza.

*Hb adduct measurements*. Hb adducts from acrylamide and glycidamide were measured in blood samples collected from 81 mothers who gave birth between 2007 and 2009 at Oslo University Hospital at Ullevål or Akershus University Hospital and enrolled in the BraMiljö and BraMat MoBa subcohorts (Gützkow et al. 2012; Stølevik et al. 2011). A common protocol for the European Commission–financed integrated project NewGeneris (Newborns and Genotoxic Exposure Risks) was followed (Merlo et al. 2009). In brief, maternal blood samples were analyzed for Hb adducts from acrylamide and glycidamide by application of the adduct FIRE procedure and analysis with liquid chromatography–mass spectrometry (Shimadzu prominence/AB Sciex 3200 qtrap; Shimadzu Corporation, Kyoto, Japan), as described by von Stedingk et al. (2010). The method performance of the adduct FIRE procedure for acrylamide and glycidamide–Hb adduct measurements has previously been described (von Stedingk et al. 2010, 2011). We excluded Hb adduct data from two women who reported smoking during pregnancy, leaving data from 79 women for comparisons with estimated dietary intakes.

*Birth outcomes and other variables*. Birth weight was measured by the midwife who attended the birth and reported to MBRN (Irgens 2000). Gestational age was calculated on the basis of first-trimester ultrasound in 98.2% of MoBa participants. In the event of a missing ultrasound measure, gestational age was calculated from last menstrual period. For births during 34–42 weeks of gestation, SGA was defined as birth weight below the 10th percentile of births among MoBa participants according to the week of gestational age at birth and parity (primiparous or multiparous). For children born during weeks 28–33 we used MBRN data published in 2000 to determine the 10th percentile of birth weight according to gestational age and parity (Skjaerven et al. 2000).

Parity was classified based on data from both MoBa and MBRN and categorized as primiparous or multiparous. Data on maternal education attainment (≤ 12, 13–16, and ≥ 17 years), age, and smoking were collected from questionnaires. Smoking during pregnancy was categorized as nonsmoker, occasional smoker, or daily smoker. Participants with unknown/missing values for education or father’s smoking were grouped in a “missing” category. Prepregnancy weight and height were self-reported at week 17 of pregnancy and used to calculate prepregnancy body mass index (BMI), which was categorized as underweight (< 18.5 kg/m^2^), normal weight (18.5–24.9 kg/m^2^), overweight (25.0–29.9 kg/m^2^), or obese (≥ 30.0 kg/m^2^). Sex of the child and weight of the mother at the time of delivery (kilograms) were collected from questionnaire 4. Maternal weight gain during pregnancy (kilograms) was calculated from weight reported at the start of pregnancy and at the time of delivery, as registered at the birth clinic on the women’s health card.

*Statistical analyses*. Spearman correlations were used to examine the relationship between dietary acrylamide intakes and measured maternal Hb adduct concentrations of acrylamide and glycidamide. Dietary acrylamide intakes were divided by total energy to derive energy-adjusted intake estimates (nanograms per kilocalorie per day) that are expressed as means ± SDs. Maternal and newborn characteristics are summarized according to quartiles of energy-adjusted acrylamide intakes and differences across quartiles were tested using chi-square or Kruskal–Wallis tests.

We used multivariable linear regression models to identify food groups that significantly predicted dietary acrylamide intakes and population characteristics associated with acrylamide intake.

We used multivariable logistic regression models to estimate associations between energy-adjusted dietary acrylamide intakes during pregnancy (categorized according to quartiles or modeled as a continuous variable) and SGA at birth, and linear regression models to estimate associations with birth weight. Potential confounders were assessed from a wide array of variables available in MoBa and were retained in all final models if they resulted in a change in estimate > 10% for either SGA or birth weight. Covariates in final models were gestational age, parity, sex of the child, age of the mother, maternal prepregnancy BMI, maternal weight gain during pregnancy, and smoking during pregnancy. Other potential covariates (type of delivery, parental education, income, father’s weight and height, marital status, and exposure to passive smoking) did not meet our criteria for confounding of associations with either SGA or birth weight, nor did maternal intakes of food groups such as snacks, sweets (including cakes and chocolate), dairy products, alcohol, or coffee, or intakes of supplements reflecting a healthy diet, such as fiber, folate, or folate supplements (data not shown).

We tested potential interactions between acrylamide intakes and active or passive smoking during pregnancy by including interaction terms in the regression models, but no significant interactions were observed (*p* for interaction > 0.10, data not shown). We confirmed that results were comparable after excluding preterm births (< 37 weeks gestation, *n* = 2,279), low birth weight children (< 2,500 g, *n* = 1,309), and women with missing information for education (*n* = 1,022) or partner’s smoking during pregnancy (*n* = 2,240) (data not shown). Results were similar when analyses were repeated using estimated acrylamide exposure relative to body weight (micrograms per kilogram body weight per day), and when the residual approach was used to adjust for energy intake or body weight (data not shown). In addition, our results were comparable after increasing or decreasing the levels of exposure in a randomly selected 20% subset as a sensitivity analysis, and when we used error-in-variables regression (Carroll et al. 1995) assuming only 80% reliability in the estimated ranking of acrylamide intake (data not shown). Data were analyzed using STATA 10.1 (StataCorp, College Station, TX, USA).

## Results

**Figure 1:**
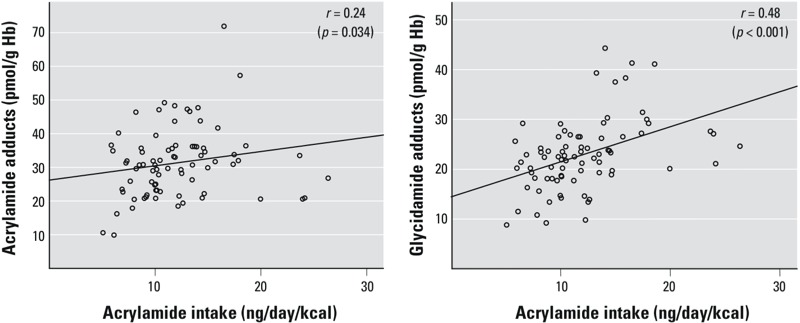
Relationships for acrylamide– and glycidamide–Hb adducts versus FFQ-based acrylamide estimated intake among nonsmoking pregnant women (*n* = 79).

*Acrylamide intake estimates and measured Hb adducts among nonsmokers*. Acrylamide– and glycidamide–Hb adducts were measured in 79 nonsmokers as markers of the internal dose of acrylamide. Mean maternal Hb adduct levels were 31 pmol/g Hb (range, 9.9–72, *n* = 79) for acrylamide and 23 pmol/g Hb (range, 8.8–44, *n* = 79) for glycidamide. A strong correlation between acrylamide– and glycidamide–Hb adduct levels (Spearman correlation = 0.62, *p* < 0.001, *n* = 79) was observed. Correlations between maternal acrylamide– or glycidamide–Hb adduct levels and energy-adjusted dietary acrylamide intakes estimated from the FFQ were 0.24 (95% CI: 0.02, 0.44) and 0.48 (95% CI: 0.29, 0.63) respectively (Figure 1).

*Acrylamide intake during pregnancy and fetal growth indicators*. Overall, the mean ± SD acrylamide intake among the 50,651 pregnant women was 27.1 ± 13.4 µg/day, 0.4 ± 0.2 µg/kg body weight/day, or 11.7 ± 4.6 ng/kcal/day. The food groups that most strongly predicted acrylamide intakes among pregnant women in the Norwegian MoBa study were snacks, which included potato chips, nuts, and popcorn; fried potatoes; and crisp bread [coefficients ± SEs were 0.17 ± 0.001, 0.15 ± 0.001, and 0.13 ± 0.001 respectively, vs. coefficients ± SEs ranging from –0.026 ± 0.0007 to 0.009 ± 0.0001 for other food groups (data not shown)].

After multivariable adjustment, increasing age, multiparity, lower educational level, maternal smoking, and paternal smoking during pregnancy were associated with significantly higher acrylamide intakes during pregnancy (Table 1).

**Table 1:** Population characteristics and dietary acrylamide intake during pregnancy.

Characteristics of acrylamide intake	n (%)	β (95% CI)		p-Value
Mother’s age (years)	30.08 ± 4.51	0.026	(0.02, 0.04)	< 0.001
Parity				
Nulliparous	26,320 (52.0)	Reference	
Multiparous	24,331 (48.0)	0.170	(0.08, 0.26)	< 0.001
Prepregnancy BMI (kg/m2)				
18.5–25	33,405 (66.0)	Reference	
< 18.5	1,437 (2.8)	0.115	(–0.13, 0.36)	0.358
25–30	11,144 (22.0)	0.063	(–0.04, 0.16)	0.216
> 30	4,665 (9.2)	–0.082	(–0.22, 0.06)	0.263
Maternal education (years)
≤ 12	15,243 (30.1)	Reference	
13–16	21,847 (43.1)	–0.205	(–0.30, –0.10)	< 0.001
≥ 17	12,539 (24.8)	–0.535	(–0.65, –0.42)	< 0.001
Missing	1,022 (2.0)	–0.513	(–0.81, –0.22)	0.001
Maternal smoking during pregnancy				
Nonsmokers	46,420 (91.7)	Reference	
Occasional smoking	2,261 (4.5)	1.181	(0.98, 1.38)	< 0.001
Daily smoking	1,970 (3.9)	1.486	(1.27, 1.71)	< 0.001
Father smoking during pregnancy				
Nonsmokers	38,325 (75.7)	Reference	
Occasional smoking	2,656 (5.2)	0.190	(0.01, 0.37)	0.041
Daily smoking	7,430 (14.7)	0.158	(0.03, 0.28)	0.011
Missing	2,240 (4.4)	0.104	(–0.10, 0.30)	0.310
Values are mean ± SD or n (%). Results are from multivariate linear regression model of energy-adjusted acrylamide intake (ng/kcal/day), adjusted simultaneously for all variables shown in the table.				

Birth weight and the frequency of SGA differed significantly by quartiles of energy-adjusted acrylamide intake during pregnancy (Table 2). The frequency of SGA was higher and the mean ± SD of birth weight was lower among women in the fourth quartile of acrylamide intake compared with women in the first quartile (11.0% and 3,591 ± 542 g vs. 9.6% and 3,612 ± 534 g respectively, *p*-values for all comparisons < 0.05).

**Table 2:** Maternal and newborn characteristics in all women and according to quartiles of energy-adjusted dietary acrylamide intake during pregnancy.

Characteristic	All women (n = 50,651)	Quartile 1a	Quartile 2a	Quartile 3a	Quartile 4a	p-Valueb
Range of acrylamide intake (g/day)		< 8.5	8.5–11.1	11.1–14.3	> 14.3
Maternal weight gain (kg)	14.9 ± 6.2	14.9 ± 6.0	14.9 ± 5.9	15.0 ± 5.9	14.9 ± 6.2	0.525
Gestational age (weeks)	39.5 ± 1.7	39.5 ± 1.9	39.5 ± 1.7	39.5 ± 1.7	39.5 ± 1.7	0.676
Birth weight (g)	3600.6 ± 539.0	3612.2 ± 533.9	3602.1 ± 538.8	3597.0 ± 541.0	3591.0 ± 541.9	0.014
SGA	5,188 (10.2)	1,216 (9.6)	1,270 (10.0)	1,311 (10.3)	1,391 (11.0)	0.003
Sex (male)	25,906 (51.1)	6,490 (51.2)	6,393 (50.5)	6,466 (51.1)	6,557(51.8)	0.224
Values are mean ± SD or n (%). aRange of acrylamide intake in ng/kcal/day. bChi-square or Kruskal–Wallis tests.						

Energy-adjusted acrylamide intake during pregnancy, modeled continuously or in quartiles, was significantly associated with SGA, with an adjusted odds ratio (OR) among all women of 1.11 (95% CI: 1.02, 1.21) for the highest quartile of acrylamide intake compared with the lowest quartile (Table 3). Associations were similar when stratified on maternal smoking (ORs for the highest versus lowest quartiles of 1.13; 95% CI: 1.03, 1.23 among nonsmokers; and 1.14; 95% CI: 0.90, 1.45 among smokers), though estimates for the smaller group of smokers were not significant.

**Table 3:** Associations between dietary acrylamide intakes (ng/kcal/day) during pregnancy and SGA.

Acrylamide intake (ng/kcal/day)	Crude			Adjusteda		
	OR (95% CI)		p-Value	OR (95% CI)		p-Value
All (n = 50,651)
Quartile 1 (< 8.5)	Reference			Reference	
Quartile 2 (8.5–11.1)	1.05	(0.97, 1.14)	0.254	1.05	(0.96, 1.14)	0.255
Quartile 3 (11.1–14.3)	1.09	(1.00, 1.18)	0.046	1.08	(0.99, 1.18)	0.061
Quartile 4 (> 14.3)	1.16	(1.07, 1.26)	< 0.001	1.11	(1.02, 1.21)	0.014
Acrylamide intake, 1-SD increase	1.05	(1.03, 1.08)	< 0.001	1.03	(1.00, 1.06)	0.029
Nonsmokers, (n = 46,420)
Quartile 1 (< 8.4)	Reference			Reference	
Quartile 2 (8.43–11.0)	1.07	(0.98, 1.17)	0.132	1.08	(0.99, 1.18)	0.092
Quartile 3 (11.0–14.1)	1.07	(0.98, 1.17)	0.132	1.09	(1.00, 1.19)	0.059
Quartile 4 (> 14.1)	1.12	(1.02, 1.22)	0.014	1.13	(1.03, 1.23)	0.008
Acrylamide intake, 1-SD increase	1.03	(1.00, 1.06)	0.056	1.03	(1.00, 1.06)	0.041
Smokers, (n = 4,231)
Quartile 1 (< 9.5)	Reference			Reference	
Quartile 2 (9.5–12.5)	1.12	(0.89, 1.41)	0.344	1.05	(0.83, 1.34)	0.655
Quartile 3 (12.5–16.0)	1.13	(0.90, 1.43)	0.288	1.06	(0.84, 1.35)	0.618
Quartile 4 (> 16.0)	1.23	(0.98, 1.54)	0.079	1.14	(0.90, 1.45)	0.263
Acrylamide intake, 1-SD increase	1.01	(1.00, 1.03)	0.081	1.01	(0.99, 1.02)	0.364
aResults from logistic regression model adjusted for gestational age, parity, sex of the child, age of the mother, maternal BMI categorical, maternal gestational weight gain (kg), and smoking during pregnancy.						

Acrylamide intake was negatively associated with birth weight, with a multivariable-adjusted coefficient of –25.7 g (95% CI: –35.9, –15.4) for birth weight for all women in the fourth quartile compared with women in the first quartile, and similar results after exclusion of smokers (Table 4). Stronger associations were observed among the smokers compared with the overall population [multivariable-adjusted coefficient –50.0 g (95% CI: –86.5, –16.6)] for the fourth quartile compared with the first quartile of acrylamide intake].

**Table 4:** Associations between dietary acrylamide intakes (ng/kcal/day) during pregnancy and birth weight (g).

Acrylamide intake (ng/kcal/day)	Crude			Adjusteda		
	β (95% CI)		p-Value	β (95% CI)		p-Value
All (n = 50,651)
Quartile 1 (< 8.5)	Reference			Reference	
Quartile 2 (8.50–11.1)	–10.1	(–23.35, 3.20)	0.137	–13.0	(–23.19, –2.81)	0.012
Quartile 3 (11.1–14.3)	–15.2	(–28.43, –1.88)	0.025	–20.8	(–31.05, –10.65)	< 0.001
Quartile 4 (> 14.3)	–21.2	(–34.44, –7.89)	0.002	–25.7	(–35.89, –15.44)	< 0.001
Acrylamide intake, 1-SD increase	–9.2	(–13.87, –4.49)	< 0.001	–9.9	(–13.50, -6.27)	< 0.001
Nonsmokers (n = 46,420)
Quartile 1 (< 8.4)	Reference			Reference	
Quartile 2 (8.4–11.0)	–11.0	(–24.82, 2.83)	0.119	–15.9	(–26.17, –4.94)	0.004
Quartile 3 (11.0–14.1)	–9.7	(–23.50, 4.15)	0.170	–19.8	(–30.55, –9.31)	< 0.001
Quartile 4 (> 14.1)	–14.4	(–28.23, –0.58)	0.041	–25.1	(–35.97, –14.73)	< 0.001
Acrylamide intake, 1-SD increase	–5.8	(–10.86, –0.92)	0.020	–9.6	(–13.48, –5.84)	< 0.001
Smokers (n = 4,231)
Quartile 1 (< 9.5)	Reference			Reference	
Quartile 2 (9.5–12.5)	–37.3	(–84.07, 9.53)	0.119	–19.1	(–55.17, 17.04)	0.294
Quartile 3 (12.5–16.0)	–45.9	(–92.66, 0.94)	0.055	–31.2	(–67.40, 5.03)	0.092
Quartile 4 (> 16.0)	–41.7	(–88.51, 5.11)	0.081	–50.0	(–86.45, –13.62)	0.007
Acrylamide intake, 1-SD increase	–12.3	(–26.96, 2.31)	0.099	–12.7	(–24.12, –1.31)	0.030
aResults from linear regression model adjusted for gestational age, parity, sex of the child, age of the mother, maternal BMI categorical, maternal gestational weight gain (kg), and smoking during pregnancy.						

## Discussion

In this study, higher maternal dietary acrylamide intakes during pregnancy were associated with evidence of impaired fetal growth based on an increase in SGA and a reduction in birth weight; similar results were obtained after excluding women who smoked during pregnancy. Dietary acrylamide intakes estimated from the MoBa FFQ were correlated with measured Hb adduct levels in a subset of maternal samples. The three food groups that most strongly predicted high intakes of acrylamide were snacks, which included potato chips, nuts, and popcorn; fried potatoes; and crisp bread. Maternal age, parity, education, and exposure to tobacco smoke also predicted the intake of dietary acrylamide during pregnancy.

Our results are in agreement with findings from a recent study of associations between fetal growth indicators and prenatal exposure to acrylamide in mother–child cohorts from five countries in Europe (Pedersen et al. 2012). Significant negative associations between maternal acrylamide exposure and birth weight among nonsmoking women were reported. Acrylamide exposure was estimated based on Hb adduct measurements in 1,101 cord blood samples, and by applying a food score approach based on the intake of acrylamide-rich foods collected by FFQs (*n* = 801).

Negative effects of prenatal exposure to acrylamide on fetal growth have been observed in animal studies with doses of a few milligrams per kilogram per day, as reviewed by Manson et al. (2005), though mechanisms are unknown. Perfusion studies have shown that acrylamide can cross the placental barrier in humans (Annola et al. 2008; Sörgel et al. 2002), and measurements of Hb adducts from acrylamide in maternal and cord blood samples have demonstrated that acrylamide is circulated in the body of the fetus (Schettgen et al. 2004; von Stedingk et al. 2011). Both acrylamide and its metabolite glycidamide are reactive electrophiles that have the potential to react with nucleophilic sites in biomacromolecules, which could affect cellular processes of importance for growth. In addition to acrylamide, other Maillard products with potential toxic effects also are formed during heat processing of foods (Chaudhry et al. 2006), and observed associations with acrylamide exposure may therefore reflect combined exposures to multiple compounds. El-Sayyad et al. (2011) reported that pregnant mice fed a diet containing 30% fried potato chips gave birth to offspring with reduced birth weight, and that the reduction in birth weight was more pronounced than expected in response to acrylamide alone, suggesting a combined effect with other compounds.

Diet is recognized as the primary source of acrylamide exposure among nonsmokers and non-occupationally exposed populations, because acrylamide is formed during cooking at high temperatures (e.g., frying, grilling, or roasting), particularly of carbohydrate-rich foods that contain the amino acid asparagine and reducing sugars (Dybing et al. 2005). The average acrylamide intake among pregnant women in our study population was 0.41 µg/kg body weight/day, which is close to previously reported intakes for a subsample of women from MoBa (0.52 and 0.44 µg/kg body weight/day based on the FFQ and a 4-day food diary, respectively) (Brantsaeter et al. 2008b), and to the median daily intake estimated in a group of nonpregnant Norwegian women 16–79 years of age (0.42 µg/kg body weight/day) (Dybing and Sanner 2003). Additionally, an FAO/WHO (Food and Agriculture Organization of the United Nations/World Health Organization) evaluation based on national survey data from 17 countries concluded that typical acrylamide intakes range from 0.3 to 0.8 µg/kg body weight/day [JECFA (Joint FAO/WHO Expert Committee on Food Additives) 2005].

Smoking was associated with higher dietary acrylamide intake in our study population. Although the interaction between acrylamide intake and smoking was not statistically significant, the association between acrylamide intakes during pregnancy and birth weight was stronger among smokers compared with nonsmokers. In contrast, the association with SGA was comparable among smokers and nonsmokers. Although tobacco smoke is the main source of exposure to acrylamide among smokers (Bergmark 1997), the added dietary burden among these women may be of concern.

The observed correlation between FFQ data and acrylamide–Hb adduct levels (Spearman correlation coefficient = 0.24) is in agreement with results reported by other investigators, whereas the correlation with glycidamide–Hb adducts (0.48) is higher than previously reported (Kütting et al. 2008; Tran et al. 2010; Wilson et al. 2009a, 2009b; Wirfält et al. 2008). Consistent with our findings, two other studies reported a higher correlation coefficient between estimated food intake and glycidamide– versus acrylamide–Hb adducts. Tran et al. (2010) reported coefficients of 0.21 and 0.16 for glycidamide– and acrylamide–Hb adducts, respectively, and Wilson et al. (2009b) reported correlation coefficients of 0.31 and 0.26, respectively. However, individual variation in the capacity to metabolize acrylamide to glycidamide would be expected to result in a weaker correlation between dietary acrylamide intakes and glycidamide–Hb adducts compared with acrylamide–Hb adducts (Duale et al 2009; Vikström et al. 2012).

The correlations between acrylamide intake estimates based on mid-pregnancy FFQs and Hb adduct levels in blood samples collected at delivery support the use of the FFQ for exposure estimation. In other studies in the general population, high within-person correlations of Hb adducts over time have been reported, suggesting that a single measurement may be a good indicator of average long-term intake (Vikström et al. 2012; Wilson et al. 2009b). The modest correlation between estimated dietary intake of acrylamide and Hb adducts indicates the uncertainty of these calculations and remains a limitation of this study. However, our results were comparable when we increased or decreased exposures by 20% in a randomly selected 20% subset, and when we used error-in-variables regression assuming only 80% reliability in the estimated ranking of acrylamide intake.

Acrylamide formation in food is affected by several parameters, such as cooking methods and doneness, that were not assessed by the FFQ (Wirfält et al. 2008). Another potential explanation for the moderate correlations between estimated dietary intakes and Hb adducts is that factors other than habitual diet may influence the biomarker. This includes exceptionally high intakes of acrylamide compared with the average diet, short time before the sampling of blood (Vikström et al. 2012), as well as cigarette smoking, which is highly associated with acrylamide–Hb adduct levels (Bergmark 1997; von Stedingk et al. 2011). Thus the FFQ-based estimate may have advantages over the biomarker because it is less likely to be affected by cigarette smoking. It is well established that both active and passive smoking are associated with a reduction in birth weight and increased risk of SGA through a range of potential mechanisms that may be independent of acrylamide (Andres and Day 2000). Thus using biomarkers that do not differentiate between exposure from smoking and exposure from dietary sources could erroneously lead to the interpretation of an effect of acrylamide intake from food that in fact could be related to smoking habits.

Selection bias, another potential concern, has been extensively explored in MoBa by Nilsen et al. (2009). On the basis of comparisons with registry data for all births in Norway, the authors concluded that although the prevalence of some characteristics among MoBa participants may show modest differences from the population as a whole (e.g., underrepresentation of women < 25 years, those living alone, or smokers), selection bias is unlikely to meaningfully influence exposure–outcome associations involving factors such as dietary supplements and smoking.

Our finding that prenatal exposure to dietary acrylamide was associated with decreased birth weight and increased SGA has implications for public health at both early and later stages of life (Barker 2002; Godfrey and Barker 2000). The associations found in this study are relatively modest. However, the estimated 25-g reduction in birth weight distribution (given the mean of 3,600 g) and the increase in the risk of SGA were statistically significant in our study population of pregnant women without abnormally high exposures. Additionally, our results support other experimental and epidemiological evidence that acrylamide-rich food is associated with a reduction in birth weight at exposure levels relevant for the general public (El-Sayyad et al. 2011; Pedersen et al. 2012).

## Conclusions

In this large population-based cohort study, higher prenatal exposure to dietary acrylamide was positively associated with SGA and negatively associated with birth weight, also after excluding smokers during pregnancy. The results suggest that prenatal exposure to dietary acrylamide may impair fetal growth. Reducing dietary acrylamide intake among pregnant women might be beneficial for fetal growth.
